# Candidate genes and molecular markers associated with heat tolerance in colonial Bentgrass

**DOI:** 10.1371/journal.pone.0171183

**Published:** 2017-02-10

**Authors:** David Jespersen, Faith C. Belanger, Bingru Huang

**Affiliations:** 1 Department of Plant Biology and Pathology, Rutgers University. New Brunswick, New Jersey, United States of America; 2 Department of Crop and Soil Sciences, University of Georgia, Griffin, Georgia, United States of America; Hainan University, CHINA

## Abstract

Elevated temperature is a major abiotic stress limiting the growth of cool-season grasses during the summer months. The objectives of this study were to determine the genetic variation in the expression patterns of selected genes involved in several major metabolic pathways regulating heat tolerance for two genotypes contrasting in heat tolerance to confirm their status as potential candidate genes, and to identify PCR-based markers associated with candidate genes related to heat tolerance in a colonial (*Agrostis capillaris* L.) x creeping bentgrass (*Agrostis stolonifera* L.) hybrid backcross population. Plants were subjected to heat stress in controlled-environmental growth chambers for phenotypic evaluation and determination of genetic variation in candidate gene expression. Molecular markers were developed for genes involved in protein degradation (cysteine protease), antioxidant defense (catalase and glutathione-S-transferase), energy metabolism (glyceraldehyde-3-phosphate dehydrogenase), cell expansion (expansin), and stress protection (heat shock proteins HSP26, HSP70, and HSP101). Kruskal-Wallis analysis, a commonly used non-parametric test used to compare population individuals with or without the gene marker, found the physiological traits of chlorophyll content, electrolyte leakage, normalized difference vegetative index, and turf quality were associated with all candidate gene markers with the exception of HSP101. Differential gene expression was frequently found for the tested candidate genes. The development of candidate gene markers for important heat tolerance genes may allow for the development of new cultivars with increased abiotic stress tolerance using marker-assisted selection.

## Introduction

Heat stress is one of the most detrimental abiotic stresses restricting the growth of cool-season plant species during the summer months. Heat stress causes changes in various physiological and metabolic processes, such as the production of reactive oxygen species (ROS) leading to oxidative damage of DNA, lipids, and proteins, altered energy metabolism resulting in a loss of carbon stores, and the degradation of proteins[1]. Plants have developed a number of mechanisms for combating heat stress, including increased antioxidant capacity to scavenge ROS, synthesis of chaperone proteins to maintain proper protein conformation, and maintenance of an active energy metabolism [1–3].

Numerous genes involved in heat tolerance mechanisms have been proposed through genomic, transciptomic, and proteomic analysis in various plant species [4–8]. The candidate gene approach has also been used to identify key genes which may control drought tolerance in various plants species, including both annual crops and perennial grasses [9–11]. Most markers developed for perennial grasses have been amplified fragment length polymorphism (AFLP), restriction fragment length polymorphism (RFLP), sequence characterized amplified regions (SCAR) or simple sequence repeat (SSR) markers used in QTL analysis to identify loci associated with disease resistance or drought tolerance [12–14]. Other marker types such as cleavage amplified polymorphic sequence (CAPS) markers or allele-specific markers have successfully been developed for candidate genes in other species to be used for QTL analysis and marker-assisted selection (MAS) for cultivar development [15, 16].

Bentgrasses, including creeping bentgrass (*Agrostis stolonifera* L.) and colonial bentgrass (*Agrostis capillaris* L.), are cool-season perennial grasses widely used as turfgrasses due to their superior canopy density and tolerance to low mowing height. Yet bentgrasses exhibit poor to moderate heat tolerance [17]. Colonial bentgrass was reported to exhibit better recuperative potential from drought damages compared to creeping bentgrass upon re-watering [18] and superior resistance to the fungal pathogen *Sclerotinia homoeocarpa* [19]. In bentgrass species, proteomic and enzymatic studies have identified a number of genes or gene products which may play important roles in heat tolerance mechanisms and may be useful as candidate genes to select for improved heat tolerance [8, 20– 22]. The identified genes included stress defense proteins such as antioxidant enzymes and chaperone proteins, as well as several related to energy metabolism.

Interspecific hybridization between colonial bentgrass and creeping bentgrass followed by backcrossing to the latter could transfer beneficial traits to creeping bentgrass [19, 23]. A colonial bentgrass x creeping bentgrass hybrid (TH15), and individuals from a mapping population created by backcrossing this hybrid with creeping bentgrass, were found to exhibit improved heat tolerance [21], drought tolerance [14], as well as disease resistance [24, 25], relative to the creeping bentgrass parental genotypes. Several QTL associated with growth and physiological traits linked to drought tolerance were detected through analysis of this creeping x colonial hybrid backcross population [14]. The availability of the backcross population provides the opportunity to determine if differences in specific genes inherited from colonial bentgrass ultimately result in phenotypic differences for improved heat stress tolerance.

The goal of this study was to develop DNA markers for candidate genes potentially associated with improved heat stress tolerance in bentgrass species. The above referenced backcross mapping population was screened for heat tolerance in addition to segregation for gene specific markers. Gene expression assays were performed on two genotypes selected from this population to help confirm the potential roles of these genes as candidate genes. Development of candidate gene markers associated with heat stress tolerance in colonial bentgrass could potentially be used for MAS in intraspecific as well as interspecific crosses in bentgrass.

## Materials and methods

### Plant materials and growth conditions

An interspecific colonial x creeping bentgrass hybrid backcross population that varied in heat tolerance was used for this study. The hybrid backcross population was created by crossing a colonial x creeping bentgrass hybrid (TH15) with a creeping bentgrass plant (9188) [24]. The population consisted of 93 individuals in addition to the hybrid parent (TH15), creeping bentgrass parent (9188), and the creeping bentgrass (5061) which was initially used to generate the hybrid (TH15).

Plants were established in pots (15 cm in diameter and 20 cm deep) filled with a mixture of 50% soil mix (fine-loamy, mixed mesic Typic Hapludult type soil) and 50% peat moss on August 24–26 2010. These plants were vegetatively propagated from population stock plants and kept in a greenhouse for 6 wk to allow for pots to establish a full and uniform canopy. Pots were moved to growth chambers (Conviron, Winnipeg, Canada) on October 5, 2010 controlled at 20/15°C (day/night) for 2 week to allow plants to acclimatize to chamber conditions before four replicate pots of each individual of the population were grown in growth chambers controlled at 20/15°C (day/night) (control) or 38/33°C (day/night) (heat stress) for 6 weeks, for a total of eight pots per genotype and a total of 768 pots including all tested genotypes and both temperature treatments. Other environmental conditions in the growth chambers included a 14-h photoperiod, photosynthetically active radiation (PAR) of 600 μmol m^-2^ s^-1^, and a relative humidity of 60%. Throughout establishment and the imposition of heat stress, plants were well watered and received half-strength Hoagland’s nutrient solution weekly [26]. The experiment was repeated in four replicates, with each temperature treatment repeated in four growth chambers for a total of eight growth chambers, with each chamber containing one entire replicate consisting of 96 pots. Pots were re-randomized among the four growth chambers every week to avoid confounding chamber effects. The experimental design was a randomized split-plot with temperature treatments as main plots and plant genotypes as subplots.

A second trial was performed on two selected genotypes, heat tolerant backcross genotype 169 and heat sensitive backcross genotype 190, from the above described population, which were found to have contrasting levels of heat tolerance. Backcross genotype 169 was consistently in the top 5% for all measured parameters and likewise backcross genotype 190 was consistently in the bottom 5%, demonstrating differences in heat tolerance levels. The trial used the same experimental set up as previously described with four replicates of the two genotypes being exposed to either non-stress control conditions for a total of 16 pots in growth chambers set to 20/15°C or heat stress conditions of 38/33°C to confirm differences in heat tolerance between the two genotypes. The second trial was initiated on January 17, 2012

### Physiological screening

Plants of the backcross population were exposed to heat stress for six weeks and were measured every two weeks for assessments of heat tolerance or stress damages which included turf quality (TQ), membrane stability, leaf chlorophyll content, photochemical efficiency, and normalized difference vegetative index (NDVI). Measurements began on October 19, 2010 with each sampling spanning two days in which all pots were sampled, subsequent sampling dates occurred on November 3, November 17, and December 1 2010. The second trial with backcross genotypes 169 and 190, heat stress was imposed for two weeks and measurements were taken weekly. Measurements began on January 17 2012 for the second trial and 1 wk and 2 wk measurements were taken on January 24 and January 31 2012 respectively. Overall plant performance based on color, density and uniformity was estimated using TQ ratings, a visual rating on a 1–9 scale with 9 being completely healthy plants and 1 being completely dead plants [27].

Membrane stability was estimated via electrolyte leakage (EL) according to the methods of Blum and Ebercon [28]. Approximately 0.1g of fresh leaf tissue was placed in a tube with 35 ml de-ionized water and shaken at room temperature for 16 h. An initial conductance reading (C_initial_) of the incubated solution was taken using a conductivity meter (YSI Incorporated, Yellow Springs, OH). Tubes were then autoclaved at 120°C for 20 min to kill all contained leaf tissue and placed back on the shaker for an additional 16 h after which time a final conductance reading was measured (C_max_). Electrolyte leakage was calculated as a percentage of C_initial_ to C_max._ to determine the percent of relative damage.

Chlorophyll content (CHL) was measured using a modification of the methods described by Hiscox and Israelstom [29]. Approximately 0.1g of fresh leaf tissue was placed in 10 ml of dimethyl sulfoxide and allowed to incubate in the dark for 5 d to extract chlorophyll from the leaf tissue. The extracted dimethyl sulfoxide solution was measured on a spectrophotometer (Spectronic Instruments, Inc., Rochester, NY) at 663 and 645 nm. Leaf tissue was then dried for 72 h in an 80°C oven to obtain dry weights and CHL was calculated on a dry weight basis using the equations described by Arnon [30].

Photochemical efficiency was estimated by chlorophyll fluorescence using a fluorescence induction monitor (Fim 1500, Dynamax, Houston, TX). Leaves were dark adapted for 30 minutes and photochemical efficiency was estimated by the ratio of variable fluorescence (Fv) to maximal fluorescence (Fm). Three subsamples per replicate were taken for each round of measurements. Normalized difference vegetative index (NDVI) estimates the green leaf biomass of the canopy based on the reflectance of red and near infrared spectral bands and can be used to estimate plant health and density [31]. A TCM 500 NDVI meter was used to measure NDVI (Spectrum Technologies, Aurora, IL) and NDVI was calculated using reflectance values (660 +/- 5 nm, 850 +/- 5 nm) and the equation NDVI = (reflectance 850 nm–reflectance 660 nm) / (reflectance 850 nm + reflectance 660 nm).

### Marker development and screening

Potential candidate genes (Table 1) involved with heat stress tolerance in bentgrasses were identified from previous research involving proteomic analysis and gene expression assays [8, 10, 32]. Allele specific markers were developed by aligning colonial bentgrass and creeping bentgrass expressed sequence tags (EST) sequences from the NCBI database for a gene of interest in the program Mega5 [33]. Exploitable polymorphisms were manually identified in the 3’ untranslated region and primers were designed to amplify only colonial bentgrass alleles. The 3’ UTR was selected due to its higher variability and polymorphisms were often identified as short indels differing between colonial and creeping bentgrasses. Primers were designed to be ~20 base pairs in length, with a Tm of 60°C, and amplify products ranging from 200–600 bp. Additionally, CAPS markers were used to identify differences in candidate genes within the population. Using the NCBI database, ESTs for genes of interest were uploaded into the program SNP2CAPS [34] which was used to identify polymorphic restriction sites. DNA was extracting using a GenElute kit (Sigma-Aldrich, St. Louis, MO) according to the manufacturer’s instructions using tissue from population individuals growing under greenhouse conditions. PCR products containing the polymorphic restriction site were amplified as described above and incubated with the restriction enzyme as per the manufacturer’s instructions. The resulting products of both allele specific markers and CAPs markers were run on a 3.5% MetaPhor (Cambrex BioScience Rockland, Inc., Rockland, ME) agarose gel and imaged using a CCD imaging system (Gel Doc XR, BioRad, Hercules CA). Table 1 contains additional information on primer sequences and marker design.

**Table 1:** Forward and reverse primer sequences, Genbank accession numbers, and marker type used for candidate gene markers.

Gene		Sequence	Accession	Marker Type
*Catalase*	F	GTGGAAGCATACTGACCCTA	DV856768	Allele Specific
	R	GGTTCATTAATCAGGGTGCATA		
*Cysteine Protease*	F	GTCCGCCATGCACACCTCA	DV858986	Allele Specific
	R	GCGTACGGAAGCAAATGTATT		
*Expansin*	F	CTCTCGCAGGAGAATCTTCA	DV853008	Allele Specific
	R	AACTGCAAGACGACGATCACACAA		
*Glyceraldehyde-phosphate-dehydrogenase*	F	CCAATGGAAGTGAACGCACG	DV856708	Allele Specific
	R	GTTCGATACTTCGAATCATTATT		
*Glutathione-S-transferase*	F	GGGCTCAACTTCTTGGCAATA	DV857137	Allele Specific
	R	CACCGAAACACACATGACACA		
*Heat shock protein 26*	F	CTGGTGGACCCGATGTCG	DV857713	Allele Specific
	R	CACGCCGTTCTTGAGCTCA		
*Heat shock protein 70*	F	ACAAGATGAAGGAGCTCGAG	DV853545	CAPS—AgsI ^a^
	R	CAGGTGATATGATTGGCACC		
*Heat shock protein 101*	F	TTCTTTCTCTGTTCGCTCTTTT	DV858769	Allele Specific
	R	CTGTTTGATTTACATGGCAATTT		

^a^AgsI restriction enzyme was obtained from Sibenzyme (Academtown, Russia) and restriction reactions were performed as per the manufacturer’s instructions.

### Real-time PCR

Candidate gene expression was analyzed in these two genotypes to support the selection of candidate genes by confirming differential expression between two genotypes contrasting in heat tolerance. Leaves of the same developmental age were harvested at 0 (January 17 2012) and 14 d (January 31 2012) of heat stress and flash frozen in liquid nitrogen for later use in gene expression assays. RNA was extracted using Trizol reagent (Life Technologies, Carlsbad, CA), treated with a Turbo DNase kit (Life Technologies) and further purification was performed with the RNeasy kit (Qiaqen, Venlo, Netherlands). A high-capacity cDNA synthesis kit (Life Technologies) was used to generate cDNA and qPCR primers were designed using PrimerExpress software (Life Technologies). A StepOnePlus real-time PCR machine was used to perform qPCR using SYBR green master mix (Life Technologies). In addition to biological replicates, three technical replicates were used per sample, along with no template negative controls. Actin was used as the endogenous control gene and relative gene expression was determined using the delta-delta-CT method [35]. The qPCR primer sequences are given in Table 2.

**Table 2:** Primers used for real-time PCR to assess candidate gene expression levels.

Gene		Sequence
*Catalase*:	F	AAGGAGAACAACTTCAAGCAG
	R	GAATCGCTCTTGCCTTGCC
*Cysteine protease*:	F	GGTTGATGAGGAACAGATTGC
	R	CAGATGTATGGGCACGACAC
*Expansin*:	F	CCCCTCTCGCAGGAGAATC
	R	TCTTGCTCAAGCTCCAAATCAA
*Glutathione-S-transferase*:	F	ACACGGTCGCCAGGTTCTT
	R	ATGACGCCGTGCAGAGGAT
*Glyceraldehyde-phosphate-dehydrogenase*	F	TCGTGGTTCAGGTCTCCAAGA
	R	GTCACGGAACGCCTGGTT
*Hsp 101*:	F	CAGCGTCATCGAGAAGGGA
	R	GACCAGGCTCTTGATCCTG
*HSP 26*:	F	GCCGTGGGACATCATGGA
	R	CTTCACTTCGTCCCGTGACA
*HSP 70*:	F	GATGGCGTTCTCTGGGTTGA
	R	GCATGGGCGGCTACATGT
*Actin*:	F	CCTTTTCCAGCCATCTTTCA
	R	GAGGTCCTTCCTGATATCCA

### Statistical analysis

Physiological data and gene expression data was assessed using ANOVA to determine significant differences between population individuals and means were separated using Fisher’s protected LSD (α < 0.05) using SAS v9.2 [36] proc GLM. Marker association to physiological parameters was performed using the non-parametric Kruskal-Wallis test (α < 0.05) using proc npar1way in SAS v9.2 [36, 37]. Kruskal-Wallis analysis is in some ways similar to a one-way ANOVA, but does not have the requirement of normal distributions. Use of this tests allowed testing for significant differences between population individuals based on the presence or absence of the candidate gene marker in an approach that is frequently used in molecular marker analysis [38, 39]. This approach was used since not all candidate genes were able to be mapped to the existing linkage map which precluded the use of an interval mapping approach. Location of markers on genetic linkage maps was determined using JoinMap 3.0 [40] using the parameters described in Rotter et al. [24].

## Results

### Genetic variation in growth and physiological traits associated with heat stress

Heat stress resulted in decline of all measurements, including TQ, EL, CHL, NDVI, and Fv/Fm, in the parents and progenies (Table 3). The average TQ score declined from 8.9 under control conditions to 5.3 by the end of heat stress; EL increased from 22.2% under control condition to 41.8% by the end of heat stress; CHL decreased by 51.6% and Fv/Fm decreased by 11.5% due to heat stress; NDVI declined from 0.33 under control conditions to 0.24 by the end of the trial.

**Table 3:** Summary of physiological measurements and ANOVA statistics from non-stress conditions and the final measurement day of heat stress for the hybrid backcross population, including parental genotypes.

	Control					6 weeks of heat stress				
Parameter	Mean	Range	Standard deviation	F-statistic	P-Value	Mean	Range	Standard deviation	**F-statistic**	**P-Value**
Turf Quality	8.9	8–9	± 0.18	2.21	0.0001	5.3	1.3–6.7	± 1.02	3.39	<0.0001
NDVI	0.33	0.22–0.47	± 0.05	2.04	0.0001	0.24	0.12–0.39	± 0.05	2.03	<0.0001
Electrolyte Leakage	22.2%	16.2%-26.2%	± 2.9	0.96	N.S.	41.8%	25.6%-96.8%	± 12.1	3.07	<0.0001
Chlorophyll Content mg/ g dw	23.7	15.12–29.2	± 2.47	2.00	0.0001	11.46	1.03–16.99	± 2.61	2.06	<0.0001
Chlorophyll Fluorescence	0.80	0.77–0.83	± 0.02	0.98	N.S.	0.71	0.46–0.76	± 0.04	1.97	<0.0001

**Fig 1:**
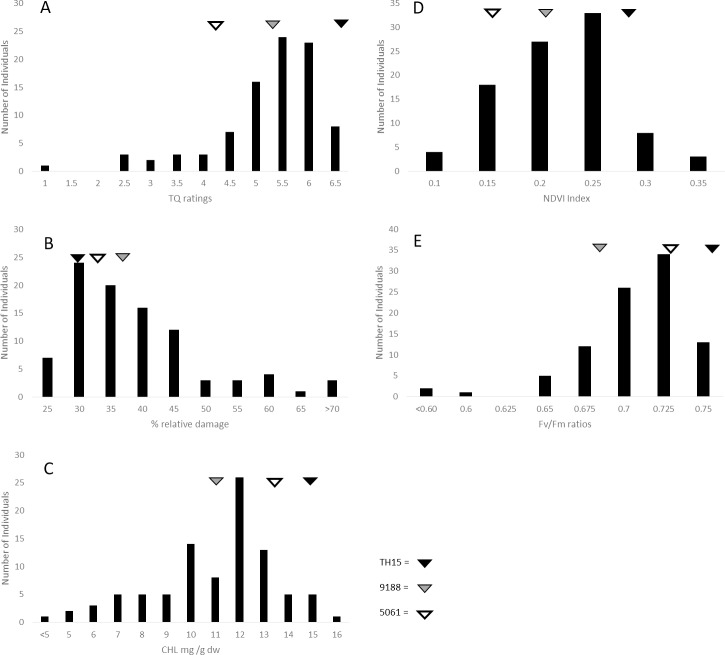
Mapping population distributions for heat stress measurements. Distributions from the final day of heat stress when differences in the population were greatest for heat stress among the 93 individuals of the mapping population for (a) TQ, (b) EL, (c) CHL, (d) NDVI, and (e) Fv/Fm. The mean values of parental genotypes are indicated with triangles, with solid black being TH15 (colonial x creeping hybrid parent), gray being 9188 (creeping parent) and white being 5061 (creeping line used to generate TH15).

The phenotypic variation within the backcross population is demonstrated by the distributions in Fig 1. Average TQ ratings for individuals of the population at the end of heat stress ranged from 1.3 to 6.7 with a standard deviation of ± 1.02; EL ranged from 25.6% to 96.8% with a standard deviation of ± 12.1; CHL ranged from 1.03 mg/g dry wt to 16.99 mg/g dw with a standard deviation of ± 2.61; Fv/Fm ratios ranged from 0.46 to 0.76 with a standard deviation of ± 0.04, and NDVI ranged from 0.12 to 0.39 by the end of heat stress with a standard deviation of ± 0.05. These data demonstrate the backcross population segregated for heat tolerance. Differences in the level of heat tolerance were found between the parents of the backcross population, with the hybrid parent being more heat tolerant than either creeping bentgrass parent, as demonstrated by significant differences in TQ, NDVI, and CHL during the trial. By the end of the trial the hybrid parent TH15 had a TQ score of 6.66, NDVI index rating of 0.27, Fv/Fm ratio of 0.773, EL of 29.7%, and CHL content level of 14.9 mg/g dw, while the creeping bentgrass parent 9188 had a TQ score of 5.33, NDVI index rating of 0.21, Fv/Fm ratio of 0.686, EL at 36.2%, and CHL content level of 10.9 mg/g dw. The creeping bentgrass 5061 had a TQ score of 4.33, NDVI index rating of 0.16, Fv/Fm ratio of 0.727, EL at 32.4%, and a CHL content level of 13.3 mg/g dw. The hybrid parent TH15 showed significantly higher TQ and NDVI relative to 5061, and TH15 had significantly better performance than 9188 for CHL at p = 0.05

**Fig 2:**
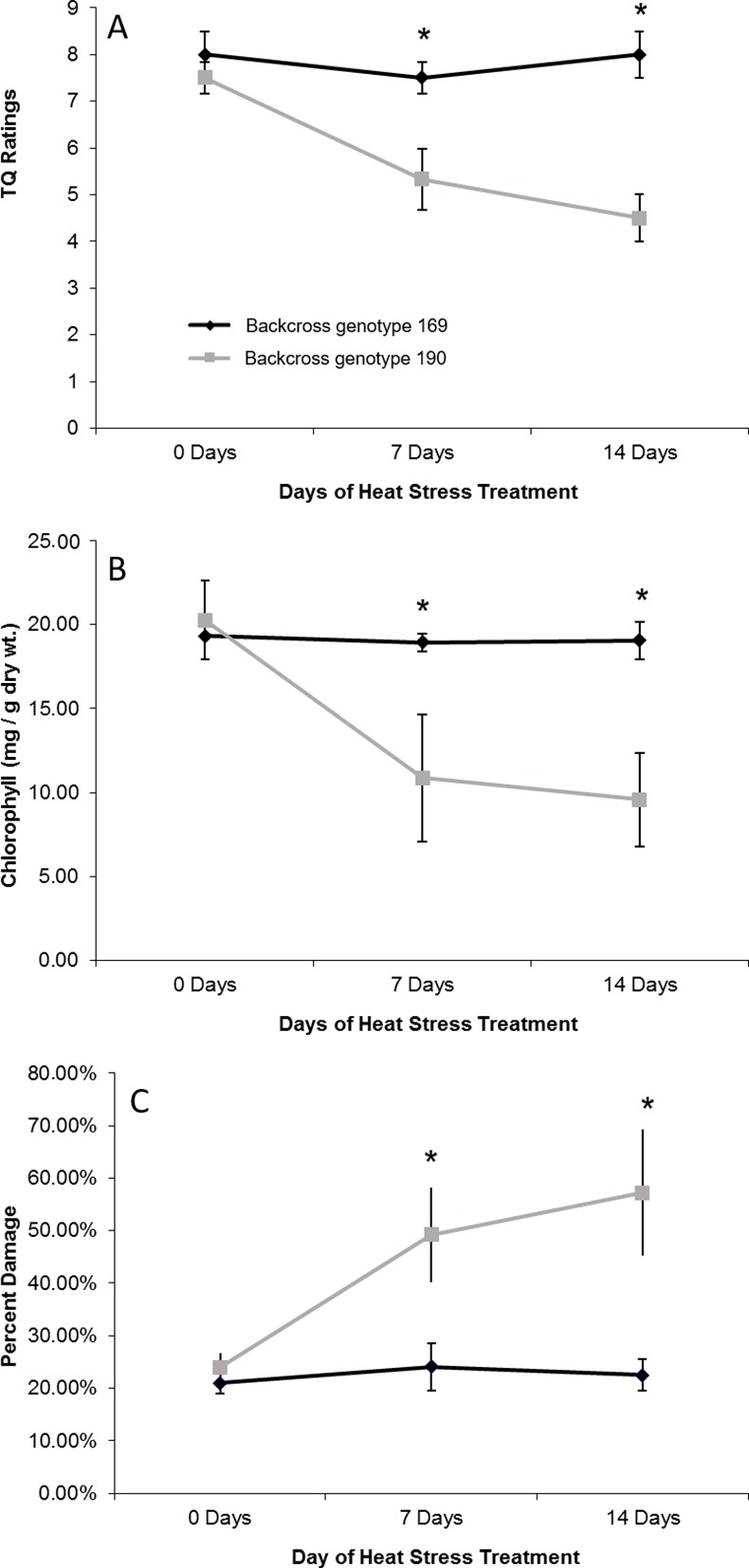
Physiological differences between two backcross genotypes contrasting in heat tolerance. Physiological differences between the heat tolerant backcross genotype 169 (black diamonds) and heat sensitive backcross genotype 190 (gray boxes) for 2 weeks of heat stress for (a) TQ, (b) CHL, and (c) EL. Bars represent standard deviations, and asterisks indicate significant differences between the two genotypes (n = 4, P < 0.05).

Two backcross individuals that varied in overall heat stress tolerance were selected for a second heat stress trial. The heat tolerant backcross genotype 169 significantly outperformed the heat sensitive backcross genotype 190 in terms of heat tolerance, as it did in the initial trial in which the whole population was tested. By two weeks of heat stress, backcross genotype 169 had maintained a TQ rating of 8 while backcross genotype 190 TQ declined to 4.5 (Fig 2A). There were no significant changes in CHL and EL by 2 weeks of heat stress for backcross genotype 169 but CHL decreased by 53% (Fig 2B) and EL increased 138% (Fig 2C) in backcross genotype 190. These results confirm that backcross genotype 169 had significantly greater heat tolerance than backcross genotype 190.

### Differential expression of selected candidate genes in response to heat stress comparing the two hybrid genotypes contrasting in heat tolerance

**Fig 3:**
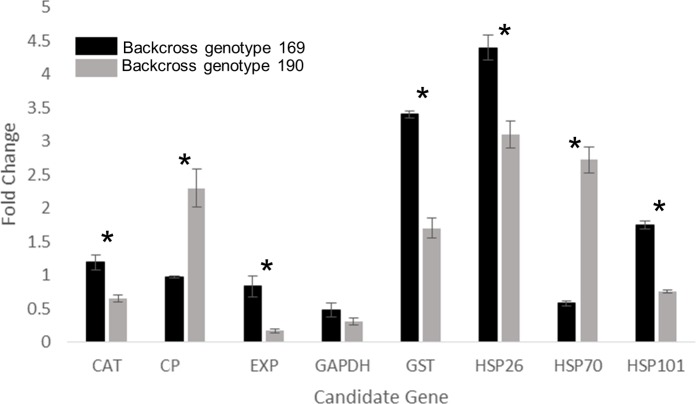
Gene expression of candidate genes in a heat tolerant and a sensitive backcross genotype. Differences in gene expression using qPCR for the heat tolerant backcross genotype 169 (black bars) and heat sensitive backcross genotype 190 (gray bars). Fold changes are changes in gene expression levels compared to control conditions (20/15°C day/night). Actin was used as the endogenous control for the delta delta CT method. Bars represent standard deviations, and asterisks indicate significant differences between the two genotypes (n = 4, P < 0.05).

Gene expression assays using real-time PCR (qPCR) were performed to determine the differential expression of selected candidate genes in response to heat stress comparing the two hybrid genotypes contrasting in heat tolerance (heat-tolerant backcross genotype 169 and heat-sensitive backcross genotype 190) (Fig 3). The transcript levels of antioxidant genes (*GST* and *CAT*) and heat-shock proteins (*HSP26*, *HSP70*, *HSP101*), cysteine protease (*CP*) for protein degradation, and expansin (*EXP*) controlling cell expansion exhibited differential responses to heat stress between the heat-tolerant and the heat-sensitive genotype when exposed to heat stress. The expression levels of *GST* (p < 0.0001), and *HSP26* (p = 0.017) significantly increased in response to heat stress, but the extent of increase differed between the heat-tolerant and the heat-sensitive genotype. The transcript levels exhibited a 3.41-fold and 1.69-fold increase for *GST*, and 1.69-fold increase and 4.39-fold increase for *HSP26* in the tolerant and the sensitive genotype, respectively. Differential expression for *HSP70* (p = 0.006) was found between the heat tolerant and sensitive genotype when exposed to heat stress. Expression levels of *HSP70* increased 2.72 fold in the sensitive genotype, but decreased by 1.72 fold in the tolerant genotype in response to heat stress when compared to the control. Conversely *HSP101* (p = 0.0002) expression levels significantly increased in the heat tolerant genotype compared to control conditions by 1.75 fold but significantly decreased by 1.32 fold in the heat sensitive genotype resulting in greater transcript levels for *HSP101* in the heat tolerant genotype. The expression levels of *CAT* and *EXP* in the heat tolerant genotype did not change significantly between heat stress and control conditions but *CAT* (p = 0.002) and *EXP* (p = 0.026) expression declined significantly by 1.53 fold and 5.68 fold when the heat sensitive genotype was exposed to heat stress resulting in significantly less expression for both genes when compared to the heat tolerant genotype. The transcript level of *CP* (p = 0.015) of the heat-sensitive genotype under heat stress had a 2.31-fold increase over control conditions, while it did not change significantly in the tolerant-genotype in response to heat stress resulting in the sensitive genotype having a significant increase in *CP* expression compared to the tolerant genotype. The transcript levels of *GAPDH* involved in energy metabolism decreased under heat stress in both genotypes when compared to control conditions, but did not significantly differ between the two genotypes.

### DNA-markers for selected candidate genes association with heat-tolerance traits

**Fig 4:**
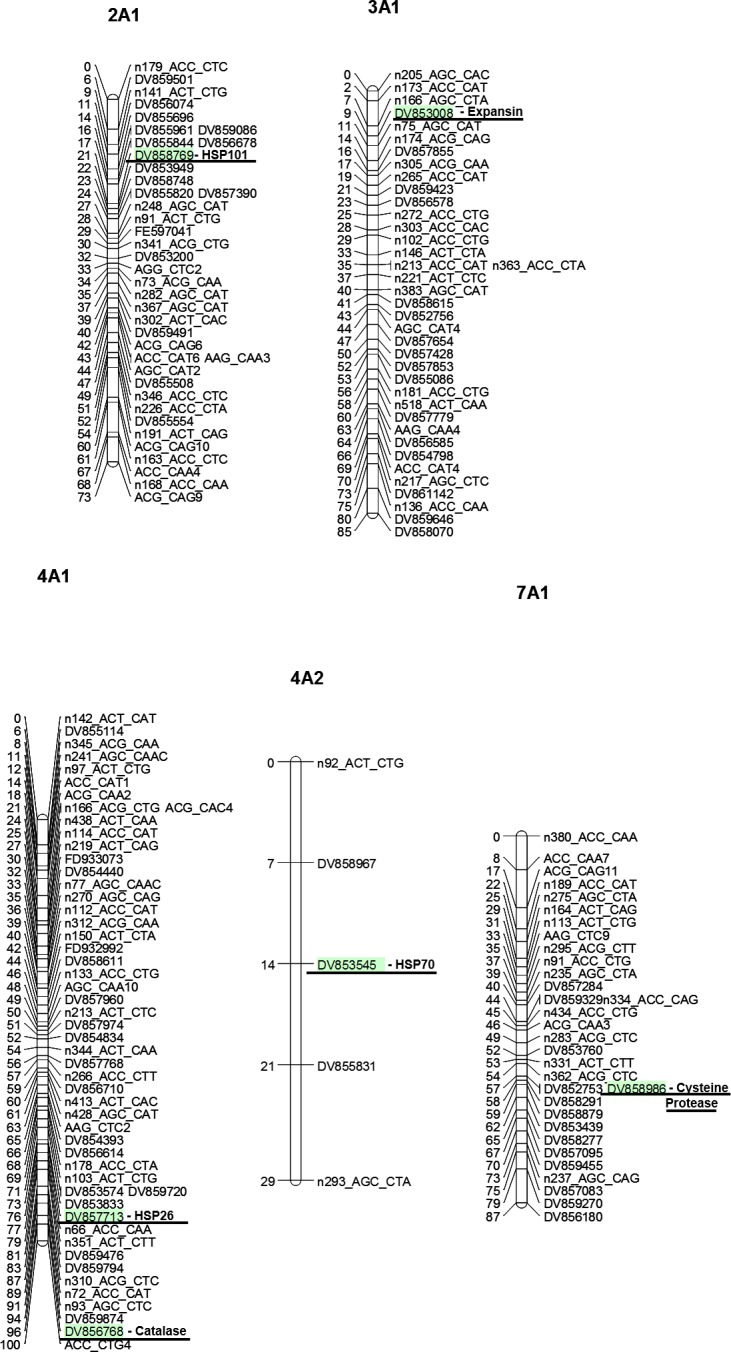
Linkage maps for colonial bentgrass with candidate gene markers. Linkage maps showing the linkage groups to which the candidate gene markers were successfully added. Added markers are highlighted and named by their accession numbers.

Candidate gene markers were mapped to linkage groups 2A1, 3A1, 4A1, 4A2, and 7A1 of the colonial bentgrass linkage map using the linkage group naming of the previously developed map [24] (Fig 4). The markers for GST and GAPDH were not successfully mapped to a linkage group, which could be due to the statistical thresholds set for adding markers to the maps or the need for greater genetic information surrounding the candidate gene markers. Presence: absence ratios for candidate gene markers among the 93 mapping population individuals were 64:29 for CP, 51:42 for EXP, 40:53 for CAT, 48:45 for GST, 19:74 for GADPH, 58:35 for HSP26, 38:55 for HSP70, and 65:28 for HSP101 (S1 Table). Kruskal Wallis analysis found significant associations between markers and the traits TQ, EL, CHL, and NDVI (Table 4). It should be noted however that it was not always the colonial bentgrass genes which provided the benefit to heat tolerance. The marker for *CP* was significantly associated with different distributions for TQ and NDVI and was positively associated with the colonial bentgrass marker. Expansin and CAT markers were both significantly associated with EL and NDVI, however this was a negative association, with the creeping bentgrass allele not the colonial bentgrass allele providing greater levels of tolerance. Similarly, genotypes with the marker for GST had significant differences in response to the photosynthetic parameter of CHL compared to genotypes which did not contain the marker according to Kurskal Wallis analysis, with the backcross population individuals who lacked the colonial bentgrass allele outperforming the individuals that did contain the colonial bentgrass allele. Both GAPDH and HSP70 markers were significantly associated with EL. The colonial bentgrass genetic background positively contributed to heat tolerance for the GAPDH marker, but was negatively associated with heat tolerance for HSP70. Genotypes with the marker for HSP26 had significantly different distributions for CHL, EL and NDVI within the population when analyzed with the Kruskal Wallis test demonstrating a significant association between HSP26 and these traits, however like the EXP and the antioxidant genes CAT and GST, the colonial bentgrass allele was negatively associated with heat tolerance. The marker for HSP101 was not significantly associated with any of the tested traits in this population.

**Table 4:** Summary of Kruskal-Wallis statistics linking candidate gene markers to physiological traits.

Candidate Gene	Physiological Trait	K statistic	Significance: P-value ^a^	Colonial association with improved tolerance	Mean of genotypes with colonial allele	Mean of genotypes without colonial allele
*Catalase*	EL	4.2	0.041	Negative	0.37	0.32
	NDVI	4.4	0.036	Negative	0.23	0.26
*Cysteine Protease*	NDVI	8.9	0.003	Positive	0.25	0.22
	TQ	6.2	0.012	Positive	5.5	5.0
*Expansin*	EL	3.9	0.042	Negative	0.32	0.30
	NDVI	3.3	0.050	Negative	0.26	0.28
*Glyceraldehyde-3-Phosphate Dehydrogenase*	EL	3.6	0.045	Positive	0.32	0.35
*Glutathione-S-Transferase*	CHL	4.5	0.035	Negative	15.5	16.7
*Heat Shock Protein 26*	CHL	7.1	0.008	Negative	15.6	16.8
	EL	4.1	0.043	Negative	0.36	0.31
	NDVI	5.3	0.021	Negative	0.24	0.26
*Heat Shock Protein 101*	-	-	N.S.	-	-	-
*Heat Shock Protein 70*	EL	3.7	0.048	Negative	0.27	0.23

^a^ Table presents the most significant data when multiple sampling days were found to be significantly associated with a given trait.

## Discussion

The mapping population used in this study exhibited a wide range of heat tolerance as demonstrated by the segregation for TQ, membrane stability estimated by EL, photosynthetic trait of CHL, and green leaf biomass measured as NDVI. Maintenance of photosynthesis and chlorophyll content, and membrane stability has been associated with improved heat tolerance in turfgrasses [41, 42]. Several traits including EL, and CHL are highly correlated with overall heat tolerance in creeping bentgrass [43]. The wide range of genetic variation in heat-tolerance traits across the creeping x colonial bentgrass backcross mapping population is of great value for the identification of candidate genes and DNA markers associated with heat tolerance.

Candidate gene markers exploited the genetic differences between colonial and creeping bentgrasses and physiological traits related to heat tolerance were at times associated with the inheritance of colonial bentgrass alleles in the backcross progeny. For several genes however it was the lack of inheritance of the colonial bentgrass alleles of the genes examined in this study that contributed to increased heat tolerance. This leads one to believe that it is the combination of genes from colonial bentgrass and creeping bentgrass background that would provide the greatest benefit for heat tolerance. The use of these or similar markers may help breeders with the introgression of colonial bentgrass genes associated with improved stress tolerance into creeping bentgrasses with desirable turf characteristics. The potential utility of an interspecific breeding program for the combination of colonial bentgrass and creeping bentgrass genes to increase stress tolerance is further supported by the hybrid parent TH15 having higher stress tolerance levels than the creeping bentgrass progenitors 9188 and 5061. The combination of both colonial and creeping bentgrass genes providing increased heat tolerance is further supported by the fact that several individual from the backcross population performed better than the hybrid parent TH15 suggesting that it is not only colonial bentgrass alleles providing all the benefit. The genetic variation in the aforementioned growth and physiological traits could be associated with candidate genes involved in serval major metabolic processes, with the colonial bentgrass alleles perhaps providing greater benefits for certain mechanisms such protection from protein degradation, and creeping bentgrass excelling in others such as antioxidant defenses. These mechanisms include cell expansion, energy metabolism and protein metabolism, as well as antioxidant defense, as discussed below.

Antioxidant metabolism is an important defense mechanism for scavenging ROS induced by stresses, including heat stress [2]. The elevated expression of antioxidant genes and the accumulation of antioxidant proteins, including peroxidase, CAT, and GST, have been positively associated with heat tolerance in various plant species, including creeping bentgrass [6, 8, 44]. A previous proteomic study found that the heat-tolerant genotype, backcross genotype 169 had a significantly greater abundance of CAT than the heat-sensitive genotype backcross genotype 190 under heat stress [21]. In this study, the heat-tolerant backcross genotype 169 had significantly higher transcript levels of both *CAT* and *GST* than the heat-sensitive backcross genotype 190 under heat stress, demonstrating the potential for *CAT* and *GST* to play positive roles in the heat tolerance of bentgrasses at the transcript level. The backcross genotypes with the candidate gene markers for the antioxidant genes, *CAT* and *GST*, had significantly different distribution patterns for EL, NDVI, and CHL. Individuals from the backcross population without the colonial bentgass alleles for both antioxidant genes had improved heat tolerance indicating that the creeping bentgrass genetic background may have had improved antioxidant capacity. The candidate genes *CAT* and *GST* were associated with increased heat tolerance, which may be of use in selecting for improved antioxidant capacity to defend against stress damages in cool-season grasses.

Heat shock proteins (HSPs) are a large family of proteins that can be induced by heat and other abiotic stresses and have been strongly linked to heat tolerance [3]. Different classes of HSPs may play various roles in stress protection, although most HSPs serve as chaperones [45]. Small HSPs, such as HSP26, are involved in stabilizing other proteins, HSP101 is involved in unfolding improperly folded proteins to aid in the return to proper conformations, and HSP70 helps to prevent protein aggregation and is involved in signal transduction [46]. Proteomic studies for bentgrasses showed an accumulation of HSPs in response to heat stress including HSP70 and HSP90 [8, 47]. In this study, the transcript levels of *HSP101* and *HSP26* were significantly greater in the heat-tolerant backcross genotype 169 compared to those in the heat-sensitive backcross genotype 190. Kruskal-Wallis analysis showed *HSP26* was associated with several physiological traits, including EL, CHL, and NDVI. However, the expression levels for HSP70 were lower in the heat tolerant backcross genotype 169. *HSP101* was significantly more up-regulated in the heat-tolerant genotype than in the sensitive genotype, but the candidate gene marker did not have significant associations with any of the physiological traits measured. Again, individuals which lacked the colonial marker for HSP26 and HSP70 showed improved heat tolerance indicating that the alleles from the creeping bentgrass materials had improved proteins for chaperone mechanisms. These results suggest that among the HSPs examined in this study, *HSP26* could play a protective role and a marker associated with *HSP26* could be valuable in developing bentgrasses with improved heat stress tolerance.

Unlike HSPs, most soluble proteins degrade or are denatured under heat stress, which disrupts various cellular functions involving protein enzymes and contributes to stress-induced leaf senescence [3, 48]. Cysteine proteases are a class of enzymes that breakdown proteins and are involved in tissue senescence [49]. A decline in soluble protein content and increases in protease activities have been observed in creeping bentgrass demonstrating symptoms of leaf senescence under heat stress [50]. In this study, the heat-tolerant genotype had a significantly lower level of *CP* expression compared to the sensitive genotype under heat stress, indicating improved heat tolerance and less senescence associated metabolism. In addition, the marker for cysteine protease was significantly associated with TQ and NDVI. These results indicated that *CP* could serve as a negative regulator for heat tolerance which is involved in the degradation of proteins during heat-induced senescence. Backcross population individuals with the colonial bentgrass allele for *CP* had improved heat tolerance, indicating that the introgression of colonial bentgrass versions of this gene may result in improved protein metabolism during heat stress or that improved heat tolerance leads to lower levels of induction of this gene during stress events. The differential gene expression of *CP* when comparing the heat-tolerant and heat-sensitive genotypes helps confirm that *CP* may be a useful candidate gene related to heat tolerance and the significant marker association with phenotypic traits suggested that cysteine protease could be used as a marker for improved breeding selection of bentgrass germplasm with enhanced heat tolerance.

Respiration metabolism play essential roles in plant adaptation to heat stress by providing energy to activate various metabolic processes [1]. One major enzyme in respiration catalyzing the breakdown processes of sugars during glycolysis is GAPDH [51]. Expression of *GADPH* has been found to be increased during heat shock conditions in Arabidopsis [52]. The protein abundance of GAPDH has been found to be positively associated with heat tolerance in creeping bentgrass plants [53]. In this study *GAPDH* expression was significantly decreased in both the tolerant and sensitive genotypes by heat stress, there was no significant difference in the level of down-regulation between the two genotypes under heat stress. However, the marker for *GAPDH* was significantly associated with membrane stability (measured as EL). The lack of significant differences in gene expression levels but significant association of a heat tolerance trait to this gene marker could be due to a number of potential factors such as post transcriptional regulation, or other factors related to the final protein product. Backcross population individuals with the GADPH gene marker had improved heat tolerance, indicating that colonial bentgrass may have improved regulation of respiration and energy metabolism resulting in improved heat tolerance. The significant marker association of *GAPDH* with membrane stability in the mapping population suggested that maintaining the supply of ATP needed for stress defense is an important mechanism for tolerance and *GAPDH* could serve as a marker to select heat-tolerance germplasm.

Expansins are a family of proteins involved in loosening cell walls and regulating cellular expansion [54]. Expansins have been found to be inducible by hormone signals in addition to being induced by abiotic stress [55]. Increased expression of expansins has been positively related to heat stress tolerance in different plant species, including creeping bentgrass [56] and wheat [5], although the exact mechanisms by which they promote heat tolerance are not fully understood. In this study, the heat tolerant genotype had significantly greater expression levels of *EXP* in response to heat stress compared to the heat-sensitive genotype, and the *EXP* gene marker was associated with membrane stability (EL) and green leaf biomass (measured as NDVI). Like several other genes in this study, it was the lack of colonial bentgrass alleles and the presence of creeping bentgrass alleles which provided the benefit to individuals. Differential gene expression between the tolerant and sensitive genotypes indicated that *EXP* could be a useful candidate gene, and significant marker associations with physiological traits suggests utility for using this marker for improving heat tolerance either through genetic transformation or molecular-assisted breeding.

In summary, the hybrid backcross mapping population showed a range of genetic variations for heat tolerance as determined by overall visual quality, NDVI, EL, Fv/Fm and CHL. Additionally when looking at candidate gene expression in a heat tolerant and heat sensitive member of the population most candidate genes had differential expression between the two genotypes under heat stress supporting that they may play roles in heat tolerance. Several candidate gene markers developed in this study (*CAT*, *CP*, *EXP*, *GAPDH*, *GST*, *HSP26* and *HSP70*) were found to be associated with important physiological traits for heat tolerance. Depending on the candidate gene being analyzed colonial bentgrass or creeping bentgrass alleles may have contributed to increased heat tolerance. The combination of specific alleles from both species may result in a turfgrass with improved heat tolerance relative to either species alone. Although further validation would be required, the successful implementation of candidate gene markers associated with heat tolerance would allow for improved selection and aid in the creation of improved turfgrass cultivars with increased heat tolerance by combining both colonial bentgrass and creeping bentgrass genes.

## Supporting information

S1 Table Segregation of candidate gene markers in population. Presence or absence of a colonial bentgrass allele for each candidate gene in the 93 individuals of the hybrid backcross population; + = Presence of the colonial bentgrass allele for a given candidate gene marker;— = absence of the colonial bentgrass allele for a given candidate gene marker.
(PDF)
