# Supplementary material for: Candidate genes and molecular markers associated with heat tolerance in colonial Bentgrass
Source: PLoS One. 2017 Feb 10;12(2):e0171183. doi: 10.1371/journal.pone.0171183 (PMC5302843; doi:10.1371/journal.pone.0171183)
Supplement: S1 Table — Presence or absence of a colonial bentgrass allele for each candidate gene in the 93 individuals of the hybrid backcross population; + = Presence of the colonial bentgrass allele for a given candidate gene marker;— = absence of the colonial bentgrass allele for a given candidate gene marker. (PDF) [file pone.0171183.s001.pdf]

Supplemental Table 1: Segregation of candidate gene markers in population. Presence or absence of a colonial bentgrass allele for each candidate gene in the 93 individuals of the hybrid backcross population; + = Presence of the colonial bentgrass allele for a given candidate gene marker; - = absence of the colonial bentgrass allele for a given candidate gene marker.

Backcross population individual	Cystein protease	Expansin	Catalse	Glutathione-S-Transferase	Glyceraldehyde-3-Phosphate DeHydrogenase	Heat Shock Protein 26	Heat Shock Protein 101	Heat Shock Protein 70
8	+	+	+	-	-	+	+	-
9	+	+	-	+	-	+	+	-
10	+	+	-	+	-	+	+	+
14	+	+	+	+	-	+	+	-
16	+	+	+	+	-	+	+	-
24	+	+	-	+	-	-	+	-
25	+	+	+	+	-	+	+	-
27	-	-	+	+	-	+	-	-
29	+	+	+	+	+	+	+	+
38	+	+	+	-	+	+	+	-
45	+	-	+	+	-	+	-	-
48	+	+	-	+	-	-	+	-
51	+	+	+	-	-	+	+	+
52	+	-	-	+	-	-	-	-
56	+	+	-	-	-	-	+	-
66	+	+	+	-	-	+	+	-
72	+	-	+	-	-	+	+	-
77	+	+	-	-	+	+	+	+
91	+	+	+	+	-	+	-	-
98	+	-	-	-	+	-	+	-
101	+	+	-	-	-	-	+	-
108	+	+	+	-	-	+	+	-
131	+	+	+	+	-	+	+	+
132	+	+	+	+	-	+	+	+
141	+	+	+	+	-	+	+	+
169	+	-	-	-	-	-	+	-
182	-	+	+	+	-	+	+	-
185	-	+	-	-	-	-	-	-
189	-	+	-	-	-	+	+	-
190	+	-	+	+	-	+	+	+
191	+	+	+	-	-	+	+	-
275	+	-	-	+	-	+	+	-
293	+	+	+	+	-	+	+	+
308	-	+	-	+	-	+	+	-
310	+	+	-	-	-	+	-	-
312	+	+	-	+	-	-	-	+
314	-	+	-	+	-	-	-	+
315	+	+	-	+	-	-	+	+
322	-	-	-	-	-	+	-	-
324	-	+	-	-	-	+	-	-

**333**	-	-	+	+	-	+	+	+
**344**	+	-	+	-	-	+	-	+
**345**	-	-	-	-	-	-	-	-
**351**	+	-	+	+	-	+	+	+
**364**	-	+	+	-	-	+	+	-
**366**	-	-	+	-	-	+	+	-
**373**	-	+	-	+	-	-	+	+
**395**	-	-	+	+	-	-	-	-
**397**	+	+	-	+	-	+	+	+
**398**	-	+	-	-	-	+	+	-
**403**	-	+	-	-	-	-	-	-
**410**	+	-	-	+	-	-	+	-
**429**	+	-	-	+	-	+	+	-
**433**	-	-	-	-	-	-	+	-
**436**	+	-	-	-	-	-	-	+
**443**	-	-	-	-	+	-	+	-
**447**	+	-	-	-	-	-	-	+
**450**	+	-	-	-	-	-	-	+
**455**	-	-	-	+	-	+	+	+
**458**	+	-	-	+	+	+	+	-
**464**	-	+	-	-	+	+	+	-
**481**	-	-	-	-	-	+	+	-
**486**	-	-	-	+	-	-	+	-
**492**	+	-	-	+	-	+	-	-
**496**	+	-	-	+	-	+	-	-
**509**	+	-	+	+	-	-	-	+
**522**	+	+	+	+	-	+	+	+
**531**	+	-	+	-	-	+	+	-
**545**	+	-	+	-	+	+	+	-
**551**	-	+	+	-	-	-	+	-
**581**	+	+	-	-	+	+	+	-
**585**	+	-	+	-	+	+	+	+
**590**	+	+	-	-	-	-	-	+
**591**	-	-	-	+	-	+	-	+
**595**	-	+	+	+	-	+	-	+
**671**	+	+	+	-	+	+	+	-
**679**	+	-	+	+	+	+	+	+
**690**	+	-	-	-	-	+	-	-
**694**	-	-	-	+	+	-	+	+
**696**	+	+	+	-	-	+	-	-
**700**	+	+	-	+	+	-	+	+
**710**	+	+	-	+	-	+	+	+
**723**	+	-	-	-	+	-	+	-
**730**	+	+	-	+	+	-	+	+
**733**	+	-	+	+	+	+	+	+
**736**	-	+	+	-	-	-	+	-
**737**	+	-	+	+	-	+	-	+
**754**	-	-	-	+	+	-	+	+

**776**	-	+	-	-	-	-	-	+
**779**	+	-	-	-	-	-	+	-
**796**	+	-	-	-	-	-	+	+
**798**	+	+	-	-	-	-	-	-
**814**	+	+	+	+	+	+	+	+

+ = Presence of the colonial bentgrass allele for a given candidate gene marker; - = absence of the colonial bentgrass allele for a given candidate gene marker.
